# Alteration of Cortical and Subcortical Structures in Children With Profound Sensorineural Hearing Loss

**DOI:** 10.3389/fnhum.2020.565445

**Published:** 2020-12-09

**Authors:** Hang Qu, Hui Tang, Jiahao Pan, Yi Zhao, Wei Wang

**Affiliations:** ^1^Medical Imaging Center, Affiliated Hospital of Yangzhou University, Yangzhou, China; ^2^College of Education, Central China Normal University, Wuhan, China; ^3^Center for Orthopedic and Biomechanics Research, Boise State University, Boise, ID, United States

**Keywords:** structural MRI, surface-based morphometry, surface-based vertex analysis, multi-modal, children, sensorineural hearing loss

## Abstract

Profound sensorineural hearing loss (SNHL) is an auditory disability associated with auditory and cognitive dysfunction. Due to distinct pathogenesis, some associated structural and functional changes within the brain have been investigated in previous studies, but whole-brain structural alterations are incompletely understood. We extended the exploration of neuroanatomic differences in whole-brain structure in children with profound SNHL who are primarily users of Chinese sign language (CSL). We employed surface-based morphometry (SBM) and subcortical analyses. T1-weighted magnetic resonance images of 26 children with profound SNHL and 27 age- and sex-matched children with normal hearing were analyzed. Compared with the normal control (NC) group, children with profound SNHL showed diverse structural changes in surface-based and subcortical analyses, including decreased cortical thickness in the left postcentral gyrus, superior parietal lobule, paracentral lobule, precuneus, the right transverse temporal gyri, and the middle temporal gyrus; a noticeable increase in the Local Gyrification Index (LGI) in the left precuneus and superior parietal lobule; and diverse changes in gray-matter volume (GMV) in different brain regions. Surface-based vertex analyses revealed regional contractions in the right thalamus, putamen, pallidum, and the brainstem of children with profound SNHL when compared with those in the NC group. Volumetric analyses showed decreased volumes of the right thalamus and pallidum in children with profound SNHL. Our data suggest that children with profound SNHL are associated with diffuse cerebral dysfunction to cortical and subcortical nuclei, and revealed neuroplastic reorganization in the precuneus, superior parietal lobule, and temporal gyrus. Our study provides robust evidence for changes in connectivity and structure in the brain associated with hearing loss.

## Introduction

Sensorineural hearing loss (SNHL) is induced by cross-modal neuroplastic changes and then associated with loss of function within the inner ear (Mutlu et al., 1998; Swanepoel et al., 2013). The incidence of SNHL each year has been estimated to be 5–20/100,000 population (Hughes et al., 1996; Olzowy et al., 2005). Also, in otologic and audiology practices worldwide, SNHL is a relatively common complaint, accounting for 1.5–1.7% of new patients (Kuhn et al., 2011). The prevalence of SNHL for children is more common than that for other congenital diseases, such as phenylketonuria and hypothyroidism (Martines et al., 2013; Swanepoel et al., 2013). These patients may show not only auditory deficits (Chilosi et al., 2010), including language delay (Yoshinaga-Itano et al., 2017), cognitive impairment (Dye and Hauser, 2014), behavioral–emotional disorders (Kronenberger et al., 2014), and impairments in posture control (Rine et al., 2004), which may elicit lifelong consequences (Shiohama et al., 2019). The total expenditure of SHNL (including nursing, special education, medications, and assistive devices) for each child with congenital severe-to-profound SNHL in the USA is >US$1,100,000 (Mohr et al., 2000).

Recently, through the use of magnetic resonance imaging (MRI), the altered structure and function of the cerebral cortex have been reported in children and adolescents with severe-to-profound or profound SNHL when compared with that of peers with normal hearing (Chilosi et al., 2010; Li et al., 2012, 2013; Shiohama et al., 2019). Surface-based cortical analyses have been used to describe several cerebral structures in auditory and non-auditory areas.

However, inconsistencies in cerebral structural changes have been documented. For instance, Li et al. (2012) found that the cortical thickness in the left precentral gyrus, right postcentral gyrus, left superior occipital gyrus, and left fusiform gyrus was significantly lower in children and adolescents with profound SNHL compared with that in the normal group. Those findings suggest that potential neuroplastic changes are linked to visual word recognition (McCandliss et al., 2003), visuo-orthographic processing (McCandliss et al., 2003), and reading skills (Desroches et al., 2010). In addition, Shiohama et al. (2019) reported a significant reduction in cortical thickness of the right superior frontal gyrus, right lateral orbitofrontal gyrus, left postcentral gyrus, left middle occipital gyrus, and left inferior occipital gyrus in children with severe-to-profound SNHL compared with that in normal hearing controls. The affected cortical areas in that study were related to visual recognition (James et al., 2003), perception of body parts and the face (Astafiev et al., 2004; Bona et al., 2015), motion stimuli (Fischer et al., 2012), and color sensitivity (Logothetis, 1999).

Those studies also emphasized the characteristics of gray-matter volume (GMV; Leporé et al., 2010; Hribar et al., 2014). Shiohama et al. (2019), using region-based and surface-based analyses, reported GMV preservation in patients with severe-to-profound SNHL (Emmorey et al., 2003; Leporé et al., 2010; Li et al., 2012; Hribar et al., 2014). Li et al. (2012) using voxel-based morphometry (VBM), showed GMV preservation in individuals with profound SNHL (Husain et al., 2011; Yang et al., 2014; Neuschwander et al., 2019; Shiohama et al., 2019). GMV comprises cortical thickness and surface area, but the proportion of each varies independently from one another (Winkler et al., 2010). Taken together, there is obvious uncertainty concerning the changes of gray matter (GM) surface area in brain regions due to a limitation of analytical methods in children with profound SNHL.

The characteristics of increased cortical folding (gyrification) have been quantified using a surface-based approach, which can be used to measure the function of sulcal depth and gyral width (Im et al., 2008; Schaer et al., 2012). Im et al. (2008) and Schaer et al. (2012) emphasized that atypical gyrification patterns reflect aberrant prenatal neurodevelopment, and are relatively stable over a lifetime. Therefore, comprehensive understanding of GM structure may yield new insights into the pathogenesis of neurodevelopmental disorders.

The diagnosis and treatment of children with SNHL has improved as a result of advanced screening and cochlear implantation (CI; Chang et al., 2012; Monshizadeh et al., 2018). Nevertheless, many continue to suffer from speech and language problems even after auditory–verbal therapy (Lü et al., 2011; Fitzpatrick et al., 2017). Consequently, sign language is used by some individuals with hearing impairment as an alternative form of communication to overcome speech and language challenges. Sign language involves use of hand movements, facial expressions, and body language to communicate (Li et al., 2014). Hence, individuals with hearing impairment who are proficient in sign language may present with a reorganized brain structure due to the reception of atypical inputs from other brain regions to compensate for the loss of auditory signals (Brookshire et al., 2017). Results from studies in patients with hearing loss who learn sign language at an early age have been very interesting (Newman et al., 2002; Li et al., 2014; Shi et al., 2016). Evidence supports a relationship between sign language and brain activation in several brain-functional areas (bilateral middle frontal gyrus, middle temporal gyrus, superior parietal lobule, superior parietal lobule, cuneate lobe, fusiform gyrus, lingual gyrus, superior temporal gyrus, inferior frontal gyrus; Newman et al., 2002; Li et al., 2014). Furthermore, surface-based cortical analyses and VBM have revealed significant differences in cortical thickness in children with profound SNHL who were trained in sign language for ≥4 years (aged 4–13 years) compared with those who were not; however, those changes were observed in non-auditory-related regions (Li et al., 2012).

Despite well-documented studies of brain alterations in children with SNHL, a lack of comprehensive and systematic research on whole-brain structural alterations persists (Emmorey et al., 2003; Leporé et al., 2010; Li et al., 2012; Hribar et al., 2014). The Local Gyrification Index (LGI) and subcortical differences in children with profound SNHL have not been investigated. Traditional morphometric methods cannot offer precise analyses of the systematic changes in brain structure or the uncertainty of the degree of brain changes involved.

Here, we employed surface-based morphometry (SBM), VBM of cortical structures, and surface-based vertex analyses of subcortical structures to investigate whole-brain structural differences in children with prelingually profound SNHL. SBM was chosen specifically because it allows measurement of additional morphometric parameters, including cortical thickness and the LGI (Schaer et al., 2012; Neuschwander et al., 2019; Shiohama et al., 2019). We hypothesized that children with profound SNHL who use sign language have significantly decreased cortical thickness, increased gyrification, and decreased volume of deep nuclei compared with that of children with normal hearing. Deeper understanding of these parameters could provide new insights into interpreting cross-modal neuroplasticity changes and compensatory mechanisms of brain activation following auditory deprivation in children with profound SNHL.

## Materials and Methods

### Ethical Approval of the Study Protocol

The study protocol was approved (2017-YKL045-01) by the Ethics Committee of the Affiliated Hospital of Yangzhou University (Yangzhou, China). Written informed consent was obtained from parents or guardians, and verbal or written consent was obtained from each child according to age-appropriateness.

### Participants

Thirty children with prelingually profound SNHL (all right-handed; SNHL group) and 30 age-, sex-, and handedness-matched normal-hearing controls (NC group) were recruited. A *post hoc* power analysis was undertaken using G*Power 3.1.7 (Faul et al., 2007). A sample size of 22 per group would have been sufficient to avoid a type-II error for our variables of interest (*p* = 80% at α = 0.001) using a two tailed *t*-test.

Prelingual deafness was evidenced by asking for the medical history of children from their family members. All participants were identified carefully based on the pure-tone audiometry test and ear examination by a very experienced otolaryngologist. The better ear of children with SNHL had a pure-tone average air-conduction threshold of 92.8 ± 2.63 dB, and the NC group demonstrated an average threshold of 15.3 ± 3.24 dB, at 500, 1,000, 2,000 and 4,000 Hz according to the Hughson–Westlake method. Children with profound SNHL were trained in Chinese sign language (CSL) that was imparted by a qualified teacher for 4.70 ± 0.62 years, and they did not have a cochlear implant. Among them, six children were treated with a hearing aid for <1 year before being trained in CSL. The NC group were monolingual Chinese speakers.

The exclusion criteria were congenital disease (e.g., heart disease, cerebral palsy, epilepsy, trisomy syndrome, hepatolenticular degeneration), psychiatric disorders, or drug abuse.

Eventually, 26 children with profound SNHL who use sign language (15 males and 11 females; 10.96 ± 0.82 years; height: 145.73 ± 5.06 cm; weight: 40.15 ± 3.58 kg) and 27 NC who were Chinese speakers (16 males and 11 females; 10.52 ± 1.25 years; 143.22 ± 6.05 cm; 40.59 ± 6.43 kg) underwent MRI and hearing assessment. Among children with profound SNHL, two cases had congenital hereditary deafness. The cause in the remainder of the cohort was viral infection, nuclear jaundice, severe otitis media, drug-induced deafness, or sudden deafness. Four children in the SNHL group and three children in the NC group were excluded from the study due to excessive head motion during MRI. Significant differences in demographic information were not detected.

### MRI

MR images were obtained using a Magnetom Verio 3.0-Tesla MRI scanner (Siemens Medical Systems, Erlangen, Germany). Children in the SNHL and NC groups were allowed to acclimatize to the environment of the MRI scanner in advance. T1-weighted anatomic images were acquired with the scan parameters of repetition time (1,900 ms), echo time (2.52 ms), inversion time (900 ms), slice thickness (1.0 mm), flip angle (9°), acquisition matrix (256 × 256) and field of vision (250 × 250 mm).

### Data Processing

#### Surface-Based Morphometry

##### Cortical Thickness and Gyrification

Data pre-processing was undertaken using the Computational Anatomy Toolbox (CAT)12 of the Statistical Parametric Mapping (SPM)12 package^1^, which were implemented in R2013b (MathWorks, Natick, MA, USA). Surface-preprocessing algorithms were used for simultaneous estimation of cortical thickness and reconstruction of the central surface of the left and right hemispheres using the projection-based thickness method (Dahnke et al., 2013). Then, the central surface and cortical thickness were estimated in one step using a projection-based distance measurement (Dahnke et al., 2013). For each participant, the data for left and right hemisphere surface-based cortical thickness were merged and smoothed with a 15-mm full-width-half-maximum (FWHM) isotropic Gaussian kernel.

LGI maps were created based on the absolute mean curvature (Luders et al., 2006). Initially, the local absolute mean curvature of the central surface was calculated by averaging curvature values from each vertex point within 3 mm for a given point. Then, LGI maps were calculated with smoothing over 20 mm (FWHM).

##### VBM Processing

Images of each participant were normalized into MNI152 space. Then, they were modulated to ensure preservation of the relative GMV after spatial normalization. These images were then smoothed with a 12-mm (FWHM) Gaussian kernel. The absolute masking threshold to the VBM data was 0.1.

To maintain quality, all images were inspected visually by experimenters before pre-processing. In addition, each image underwent a statistical quality control for inter-person homogeneity and overall image quality as included in the CAT12 toolbox (“check homogeneity” function) after segmentation (Supplementary Figure 1).

#### Subcortical Nuclei

Subcortical nuclei were processed using the FIRST segmentation algorithm of FSL^2^ (run_first_all script; Supplementary Figure 2). The whole brain was registered as a T1 image to the MNI152 standard space using a nonlinear template. Shape variance was reserved after registration. Then, subcortical nuclei were registered to an MNI152 subcortical mask. After alignment, meshes were generated by a deformable model that updated the vertex locations iteratively according to a weighted sum of displacements (Patenaude et al., 2011). Then, localized changes in shape were compared directly by analyzing vertex locations (which considered differences in the mean vertex position between groups). The quality of segmentation was checked visually by two independent experimenters.

### Statistical Analyses

Statistical analyses of imaging data were undertaken in the CAT12 surface-based linear model by applying two-sample *t*-tests to each of three morphometric measurements (cortical thickness and gyrification with SBM, and GMV with VBM). Using age and sex as covariates (and, additionally for VBM analyses, total intracranial volume), group differences were tested applying thresholds of *p* < 0.05 with a Threshold-Free Cluster Enhancement (TFCE; Salimi-Khorshidi et al., 2011) correction for multiple comparisons. Statistical analyses of the shapes of subcortical nuclei were conducted using the “randomize” module in FSL (Winkler et al., 2014). A general linear model, with a two-sample *t*-test design, was employed to compare differences in the shapes of subcortical nuclei between the two groups. Multiple comparisons were corrected at the cluster level using a TFCE, with a family-wise error rate of *p* < 0.05 (the number of permutation tests was set at 5,000). Differences in volumes of subcortical nuclei between groups were compared using analysis of a nonparametric rank sum test carried out in SPSS 17.0 (IBM, Armonk, NY, USA), with age and intracranial volume (ICV) as covariates.

## Results

### Surface-Based Morphometry

**Figure 1:**
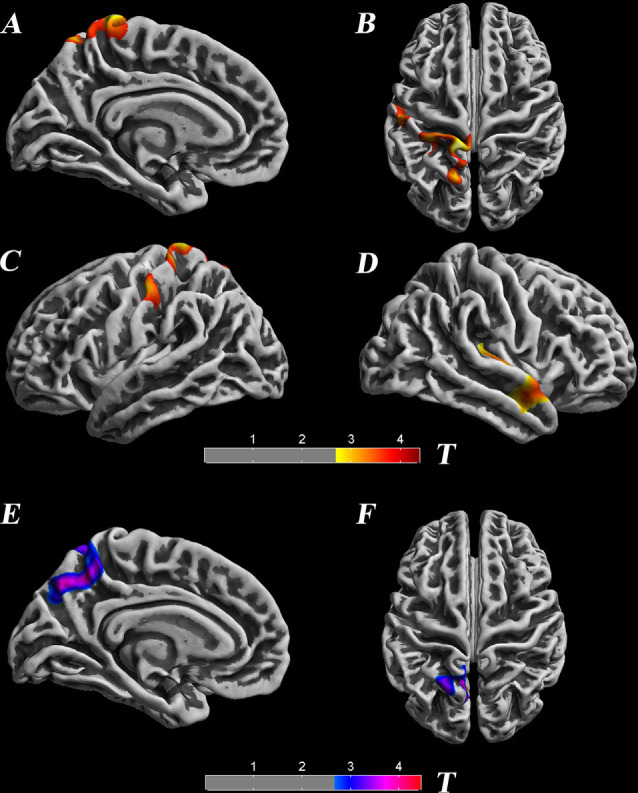
Significant differences in the cortical thickness between groups [*p* < 0.05, threshold-free cluster enhancement (TFCE) corrected], which are highlighted with significance-levels visualized on a red to yellow scale and superimposed on a template of a reconstructed brain surface in MNI space. Decreased thickness was detected in the left postcentral gyrus, superior parietal lobule, paracentral lobule, precuneus, right transverse temporal gyrus, and middle temporal gyrus **(A–D)**. Moreover, local gyrification index (LGI) differences are highlighted with significance-levels visualized on a blue to purple scale (*p* < 0.05, TFCE corrected). A noticeable increase in LGI was found in the left precuneus and superior parietal lobule areas **(E,F)**.

Surface-based morphometry showed that cortical thickness and the LGI were significantly different between SNHL and NC participants (Table 1, Figure 1). Decreased thicknesses were detected in the left postcentral gyrus, superior parietal lobule, paracentral lobule, precuneus, right transverse temporal gyrus, and middle temporal gyrus. A noticeable increase in the LGI was found in the left precuneus and superior parietal lobule areas (*p* < 0.05, TFCE-corrected).

**Table 1:** Surface-based cortical measurements in SNHL and NC groups (*p* < 0.05 TFCE whole-brain corrected with corresponding cluster size and proportion).

		Cluster location	Size (number of vertex)	Mean cortical thickness in SNHL group (mm)	Mean cortical thickness in NC group (mm)	Proportion of the cluster in this brain region	*t*-values
Cortical thickness	Cluster1	Left postcentral	3,130	2.62 ± 0.14	2.85 ± 0.19	52%	4.5
		Superior parietal	-	-	-	24%	-
		Precuneus	-	-	-	13%	-
	Cluster2	Left postcentral	1,020	2.74 ± 0.21	2.93 ± 0.18	100%	3.4
	Cluster3	Right superior temporal	2,592	2.72 ± 0.13	2.90 ± 0.15	61%	4.0
		Transverse temporal	-	-	-	26%	-
Gyrification index	Cluster4	Left precuneus	3,225	-	-	62%	4.0
		Superior parietal	-	-	-	38%	-

*SNHL, Sensorineural hearing loss; NC, Normal control; TFCE, threshold-free cluster enhancement. Only significant group differences are shown in this table*.

### Voxel-Based Morphometry

**Figure 2:**
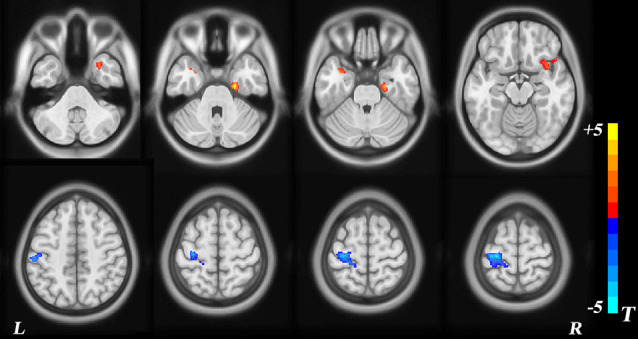
Voxel-based morphometry (VBM) analysis of group differences. The red area represents a modulated gray-matter volume (GMV) increase in children with sensorineural hearing loss (SNHL), with blue representing a decrease.

VBM morphometry revealed group differences in regional GMV. Decreased modulation of GMV was detected in the left postcentral, left precentral, and left inferior parietal regions. Increased modulation of GMV was observed in the right temporal pole, right superior temporal gyrus, right insula, right inferior temporal gyrus, left temporal pole, left superior temporal gyrus, and right parahippocampal regions (*p* < 0.05, TFCE-corrected; Table 2, Figure 2).

**Table 2:** Voxel-based morphometry measurements in SNHL and NC groups (*p* < 0.05 TFCE corrected for multiple comparisons).

Area	MNI coordinates (mm); *X Y Z*	Cluster size	Mean modulated GMV in SNHL group	Mean modulated GMV in NC group	*t*-value
Left postcentral, precentral	–30–37 72	1,096	0.35 ± 0.02	0.41 ± 0.03	−4.9
Left postcentral, inferior parietal	–55–24 48	165	0.36 ± 0.02	0.41 ± 0.04	−4.2
Right temporal pole, superior temporal gyrus, insula	46 24–21	255	0.49 ± 0.04	0.47 ± 0.06	3.7
Left temporal pole, superior temporal gyrus	–30 3–33	147	0.41 ± 0.04	0.39 ± 0.05	4.2
Right inferior temporal gyrus	28 12–40	148	0.40 ± 0.04	0.38 ± 0.05	3.7
Right parahippocampal	22–13–34	217	0.41 ± 0.05	0.39 ± 0.05	4.6

*Coordinates of primary peak locations in the MNI space. SNHL, Sensorineural hearing loss; NC, Normal control. Only significant group differences are shown in this table*.

### Surface-Based Vertex Analyses

**Figure 3:**
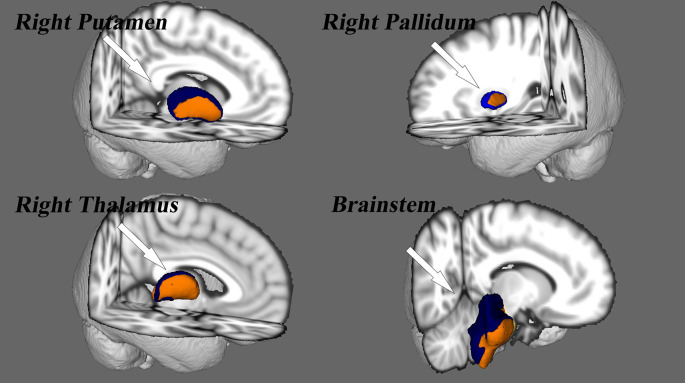
Vertex analysis for the subcortical nuclei. Nuclei deformity are color-coded by *t*-statistic value thresholds, corrected by FWE at a rate of *p* < 0.05.

Vertex-wise shape (VWS) analyses revealed significant regional contractions in subcortical nuclei, including the right pallidum, putamen, thalamus, and brainstem [*p* < 0.05, Family Wise Error (FWE) corrected; Figure 3]. Other subcortical nuclei did not show differences in regional shape between SNHL and NC groups. The right thalamic volume (*p* = 0.04) and right pallidum volume (*p* = 0.02) were decreased significantly in the SNHL group (Supplementary Table 1).

## Discussion

The purpose of the present study was to examine, using SBM and vertex analyses, structural alterations in the brain anatomy of children with profound SNHL who use sign language. In accordance with our hypothesis, SBM analyses detected reduction of cortical thickness in the left postcentral gyrus, superior parietal gyrus, paracentral lobule, right transverse temporal gyrus, and superior temporal gyrus, as well as an increased LGI in the left precuneus and superior parietal gyrus. A modulated decrease in GMV occurred in the left postcentral, precuneus, and inferior parietal lobule, whereas increased GMV was observed in the bilateral temporal pole, superior temporal gyrus, and the parahippocampal area, findings which are not in accordance with our hypothesis. Surface-based vertex analyses showed regional contractions in the right pallidum, putamen, thalamus, and brainstem nuclei. In addition, the right thalamus and pallidum nuclei exhibited smaller volumes in children with SNHL, findings that also support our hypothesis. Therefore, these results are partly consistent with our original hypothesis.

SBM showed structural alterations in auditory and non-auditory cortices in children with profound SNHL. Studies reporting a reduction of cortical thickness in the postcentral gyrus (Li et al., 2012) and superior parietal gyrus (Shiohama et al., 2019) of children with severe-to-profound SNHL were consistent with our research results. These regions are the primary somatosensory cortices related to processing motion stimuli. However, differences in cortical thickness have also been found in occipital areas, such as the left-middle and inferior occipital (Shiohama et al., 2019) and left-superior occipital gyri (Li et al., 2012), which are involved with visual recognition (James et al., 2003). Scholars have postulated that such differences are the result of a transmodal neural change, which reflects the adaptability and compensation of brain function. That is, because the input of the central auditory system is severely reduced or lacking, other sensory inputs would be enhanced correspondingly (Lomber et al., 2010). However, in our study, a significant change observed in the visual cortex was not observed; alternatively, the higher-order auditory cortex appeared to be recruited by somatosensory and sensorimotor modalities in childhood deafness. Conversely, most studies have not reported differences in the region of the auditory cortex in deaf children who are not auditory verbal communicators or signers (Li et al., 2012; Shiohama et al., 2019), data which are in contrast with our results.

Notably, cortical thickness on the left precuneus was found to be decreased, whereas the LGI was increased significantly. Gyrification is an important property of the cortex and helps to increase the surface area of the brain within a finite space, and may be related to general cognitive abilities (Docherty et al., 2015). Furthermore, the precuneus responds to a wide range of cognitive processes (Zhang et al., 2015), such as awareness and conscious information processing (Vogt and Laureys, 2005), episodic memory (Dörfel et al., 2009), and visuospatial processing (Wenderoth et al., 2005). In addition, deaf children have to orient to new incoming information through vision, monitor peripheral visual fields (which enhances neural responses) and identify hand motion through improved motion sensitivity that arises with the acquisition of sign language (Bavelier et al., 2001). Such children might need to integrate all this information to overcome challenges related to speech and language. Given this information, we believe that the observed increase in the LGI may be related to the need for information integration and cortical contacts in children with SNHL. Taken together, these alterations suggest that connectivity with the auditory–visual cortex and auditory–somatosensory cortex may be changed by hearing loss due to adaptive and compensatory processes that originate from cross-modulated plasticity within the auditory cortex.

VBM revealed a decrease in GMV of the left postcentral, precentral, and inferior parietal gyrus, which correspond to somatosensory cortex, primary motor cortex, and sensory speech areas, respectively. Smaller GMV in the left postcentral, precentral, and inferior parietal gyrus correlates with poor attention (Li et al., 2010), low ability in various motor functions (e.g., movement of contralateral limbs, contralateral movement of the face and mouth; Qiu et al., 2019), and impaired semantic processing in sentence comprehension (Kambara et al., 2013). Those data suggest that hearing impairment may result in overall dysfunction of motor and language skills. In the current study, increased GMV was observed in bilateral temporal poles, superior temporal gyrus, and parahippocampal area. The parahippocampal gyrus is a cortical region surrounding the hippocampus, and it is part of the limbic system that plays an important part in the encoding and retrieval of memory. Adjacent areas in the superior and lateral portions of the temporal lobes are involved in high-level auditory processing. The ventral part of the temporal cortices appears to be involved in high-level visual processing of complex stimuli, such as faces (fusiform gyrus) and scenes (parahippocampal gyrus). The anterior parts of this ventral stream for visual processing are involved in the perception and recognition of objects (Hickok and Poeppel, 2007). The increased GMV observed in these areas may be related to the integration and remodeling of audiovisual function in children with SNHL.

Our study results are in agreement with those of Li et al. (2012), who found no significant changes in GMV of the left-middle frontal gyrus and right-inferior occipital gyrus in children with profound SNHL when compared with that of a hearing group (Li et al., 2012). The left-middle frontal gyrus is the specific area for reading of Chinese text (Siok et al., 2003), and right-inferior occipital gyrus is responsible for visual processing of Chinese text (Siok et al., 2004). Hence, we suggest that a lack of structural decrease in these areas may be associated with reading of Chinese text (Li et al., 2012). In the current study, children with SNHL could not read Chinese very well based on their teachers’ evaluation. However, they possessed great skills in the production and comprehension of sign language. These children’s early exposure to sign language might lead to greater reliance on cerebral organization for processing of language and motion (Bavelier et al., 2001). Further comparisons between the results of our study from that of research with adults have shown increased GMV in the structures involved in motor processing (i.e., precentral area) in native deaf signers (Leporé et al., 2010; Olulade et al., 2014), but no differences in adult deaf signers (Li et al., 2015). Congenitally deaf signers may use sign language more frequently and, thus, would be more proficient than postnatal deaf signers, which may have assisted in the observed structural remodeling. In addition, reports have described that normal brains undergo dramatic structural change until adolescence; specifically, the auditory network of neurofilament-positive axons in superficial layers IIIb, IIIa, and II mature from 5 to 12 years of age (Moore and Guan, 2001). Given that the ages of participants in our study ranged from 9 to 13 years, children with SNHL might be slow to show various GMV changes during this time due to natural reorganization of auditory areas (Tae, 2015).

Alterations in subcortical structures in children with auditory deprivation were also observed in our study. VWS analyses demonstrated significant regional contractions of the right thalamus, pallium, putamen, and brainstem in the SNHL group. The thalamus has a critical role in the relay and integration of sensory afferences and motor efferences to and from the auditory cortex (Tae et al., 2018; Simon et al., 2020). In addition, primary thalamo–auditory connections originate in the medial geniculate complex, and are often involved in auditory thalamic exchange (Hackett, 2011). The reduction in volume observed in the right thalamus of children with SNHL align (at least in part) with diffusion tensor imaging studies reporting a reduction of fractional anisotropy in fibers projecting to the right internal capsule next to the thalamus in deaf individuals (Hribar et al., 2014; Lyness et al., 2014; Olulade et al., 2014). However, a study by Amaral et al. (2016) on asymmetries in subcortical structures in congenital deafness found that the right thalamus, right lateral geniculate nucleus, and right inferior colliculus were larger than their left-sided counterparts. These findings may be related to the asymmetry of brain development and may explain why we found only lateral changes in various subcortical structures. We also noticed differences in the right putamen and pallidum areas. This may have been because basal ganglia are involved with involuntary motor activity and muscle tone in these areas. This hypothesis is supported by studies reporting reduction of WM in the right hemisphere of deaf adults who used sign language exclusively to communicate (Meyer et al., 2007). Furthermore, the inferior colliculus is the main nucleus of the auditory system that receives ascending signals from inferior brainstem nuclei (Tae et al., 2018). Hence, the significant finding within the brainstem of children in the SNHL group in our study is reasonable. Taken together, these results suggest that auditory deprivation may be related to subcortical cross-modal neuroplastic changes.

Our study had three main limitations. First, due to the small sample size, narrow age range of participants, and the lack of clear diagnostic evidence, we could not classify participants into specific subgroups, which may have influenced the results. In the future, we will explore the correlation between structural alterations of different causes and SNHL. Second, although we observed structural alterations in cortices and subcortices, the complete nuclear connectivity of the auditory area is not known. Third, we excluded children who underwent CI, which may have resulted in incomplete interpretation of structural changes of the brain in children with profound SNHL. In future studies, we will conduct regular follow-ups, monitoring, and evaluation of children so that the longitudinal dynamic changes in brain structure can be examined.

## Conclusion

We explored structural alterations in the brains of 26 children with profound SNHL using surface-based structural MRI. Children with profound SNHL showed reduced cortical thickness in several regions, an increased LGI, decreased GMV, and alterations in subcortical structures. These results provide empirical evidence for understanding the neuroplastic mechanisms and compensatory brain reconstruction underlying auditory deprivation. Our data could aid use of potential biomarkers for monitoring the progression of cortical and subcortical structures for children with SNHL.

## Data Availability Statement

The original contributions presented in the study are included in the article/Supplementary Materials, further inquiries can be directed to the corresponding author.

## Ethics Statement

The studies involving human participants were reviewed and approved by Ethics Committee of the Affiliated Hospital of Yangzhou University (approval number 2017-YKL045-01). Written informed consent to participate in this study was provided by the participants’ legal guardian/next of kin.

## Author Contributions

HQ, HT, and WW designed the research. HQ, HT, and YZ undertook the research and analyzed the data. HT and JP wrote the manuscript. All authors contributed to the article and approved the submitted version.

## Conflict of Interest

The authors declare that the research was conducted in the absence of any commercial or financial relationships that could be construed as a potential conflict of interest.

## Footnotes

^1^http://dbm.neuro.uni-jena.de/cat12/

^2^https://fsl.fmrib.ox.ac.uk/

## Supplementary Material

SUPPLEMENTARY FIGURE 1 Image segmentation quality control.

SUPPLEMENTARY FIGURE 2 Segmented using the run_first_all script.

SUPPLEMENTARY TABLE 1 The volumetric analysis measurements in SNHL and NC group.
