# Supplementary material for: Alteration of Cortical and Subcortical Structures in Children With Profound Sensorineural Hearing Loss
Source: Front Hum Neurosci. 2020 Dec 9;14:565445. doi: 10.3389/fnhum.2020.565445 (PMC7756106; doi:10.3389/fnhum.2020.565445)
Supplement: SUPPLEMENTARY FIGURE 1 — Image segmentation quality control. [file Data_Sheet_1.PDF]

Supplementary Material

Supplementary Table

The volumetric analysis measurements in SNHL and NC group.

	right thalamic volumes	right pallidum volumes
**SNHL group**	**7847.8 ±535.42**	**1667.06 ±137.58**
**NC group**	**8186.62 ±789.15**	**1757.44 ±145.74**

Supplementary Figures

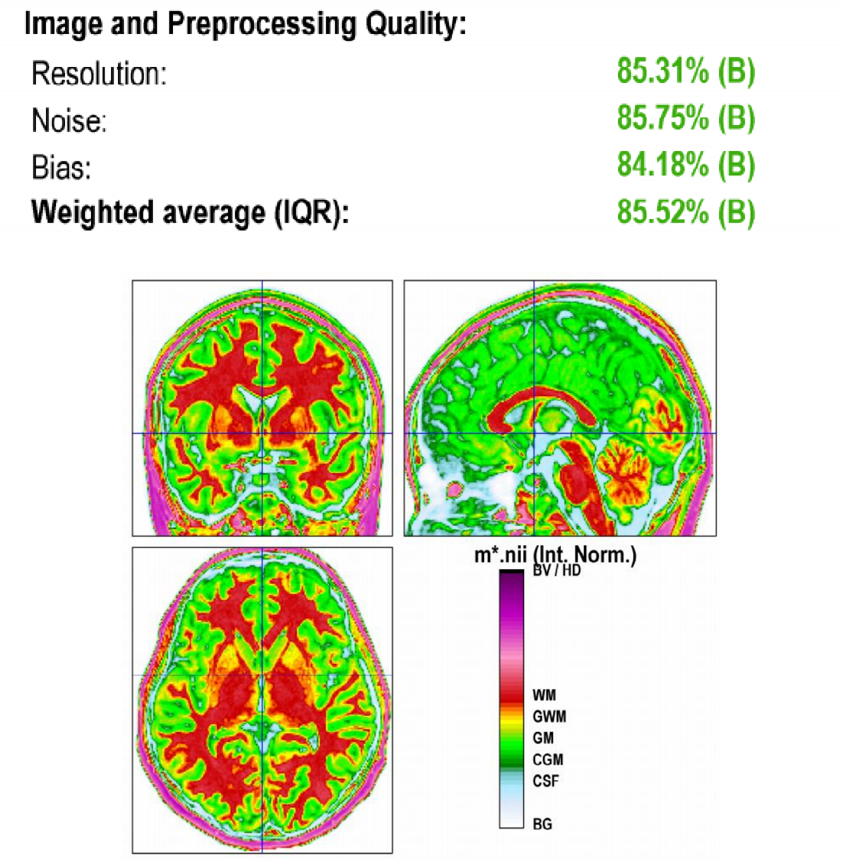

Supplementary Figure 1. Image segmentation quality control.

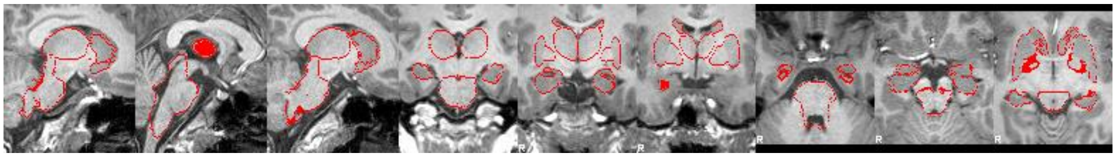

Supplementary Figure 2. Segmented using the run_first_all script.
